# Promoting professional behaviour change in healthcare: what interventions work, and why? A theory-led overview of systematic reviews

**DOI:** 10.1136/bmjopen-2015-008592

**Published:** 2015-09-30

**Authors:** Mark J Johnson, Carl R May

**Affiliations:** 1 National Institute for Health Research, Southampton Biomedical Research Centre, University Hospital Southampton NHS Foundation Trust, and University of Southampton, Southampton, UK; 2 University Hospital Southampton NHS Foundation Trust, Southampton, UK; 3 Faculty of Health Sciences, University of Southampton, Southampton, UK

**Keywords:** Professional practice, Behaviour, Health Services, Implementation

## Abstract

**Objectives:**

Translating research evidence into routine clinical practice is notoriously difficult. Behavioural interventions are often used to change practice, although their success is variable and the characteristics of more successful interventions are unclear. We aimed to establish the characteristics of successful behaviour change interventions in healthcare.

**Design:**

We carried out a systematic overview of systematic reviews on the effectiveness of behaviour change interventions with a theory-led analysis using the constructs of normalisation process theory (NPT). MEDLINE, CINAHL, PsychINFO and the Cochrane Library were searched electronically from inception to July 2015.

**Setting:**

Primary and secondary care.

**Participants:**

Participants were any patients and healthcare professionals in systematic reviews who met the inclusion criteria of having examined the effectiveness of professional interventions in improving professional practice and/or patient outcomes.

**Interventions:**

Professional interventions as defined by the Cochrane Effective Practice and Organisation of Care Review Group.

**Primary and secondary outcome measures:**

Success of each intervention in changing practice or patient outcomes, and their mechanisms of action. Reviews were coded as to the interventions included, how successful they had been and which NPT constructs its component interventions covered.

**Results:**

Searches identified 4724 articles, 67 of which met the inclusion criteria. Interventions fell into three main categories: persuasive; educational and informational; and action and monitoring. Interventions focusing on action or education (eg, Audit and Feedback, Reminders, Educational Outreach) acted on the NPT constructs of Collective Action and Reflexive Monitoring, and reviews using them tended to report more positive outcomes.

**Conclusions:**

This theory-led analysis suggests that interventions which contribute to normative restructuring of practice, modifying peer group norms and expectations (eg, educational outreach) and relational restructuring, reinforcing modified peer group norms by emphasising the expectations of an external reference group (eg, Reminders, Audit and Feedback), offer the best chances of success. Combining such interventions is most likely to change behaviour.

**Strengths and limitations of this study**

- This overview of systematic reviews of professional behaviour change interventions investigates heterogeneous, non-standardised and complex interventions and provides indicative rather than definitive conclusions about effectiveness.
- This overview of systematic reviews identifies the types and combinations of interventions more likely to successfully initiate and sustain professional behaviour change in the context of complexity, which may not have been captured by a standard systematic review.
- This overview explains the relative strengths and weakness of different intervention types using a rigorous theoretical framework, highlighting mechanisms common to the most effective interventions.

## Introduction

Finding effective ways to encourage health professionals to routinely embed high-quality clinical evidence into their everyday work is important, but has proved a major challenge.^1^ The past 20 years has seen a very significant international programme of research and development that aims to meet this challenge. There is now a voluminous literature, reporting many clinical trials and systematic reviews of professional behaviour change interventions in many different settings. How these interventions are characterised and defined has been shaped in important ways by the methodological programme of the Cochrane Effective Practice and Organisation of Care (EPOC) Review Group.^2^ Their robust set of definitions has included a taxonomy of professional interventions (described in table 1), and has been an important scientific innovation because it has meant that researchers have a methodological vocabulary that enables a shared understanding of intervention types and evaluation procedures. This has led to a focus on achieving very high levels of precision in intervention design and testing, and an emphasis on explanations of intervention take-up that has often modelled professional behaviour change as a feature of agents working relatively autonomously. Medical professionals—and especially family doctors—have been an important focus of such work. However, most professional behaviour change interventions are now ‘complex interventions’ that are operationalised in complex organisational and policy contexts.^3^ This means that many of the traditional approaches to understanding professional behaviour change—for example, social cognitive theories that emphasise the importance of individual attitude→intention processes,^4^ or principal-agent and other economic theories that emphasise individual self-interest and promote financial incentives^5^ ^6^—may be less useful than previously supposed in explaining behaviour change and characterising its underlying processes. This is because complex interventions in complex settings tend to be implemented through collective action that takes place when people work together, rather than as a result of individual behavioural processes.^7–9^ Context is important: these interventions encompass a wide range of behaviours—from hand washing in hospitals to medication management in primary care—across many different kinds of national healthcare systems, healthcare provider organisations and within and between diverse professional groups.

**Table 1:** Professional interventions as per Cochrane EPOC review group (adapted from^2^)

	Name	Description
A	Distribution of educational materials	Distribution of published or printed recommendations for clinical care, including clinical practice guidelines, audiovisual materials and electronic publications. The materials may have been delivered personally or through mass mailings
B	Educational meetings	Healthcare providers who have participated in conferences, lectures, workshops or traineeships
C	Local consensus processes	Inclusion of participating providers in discussion to ensure that they agreed that the chosen clinical problem was important and the approach to managing the problem was appropriate
D	Educational outreach visits	Use of a trained person who met with providers in their practice settings to give information with the intent of changing the provider's practice. The information given may have included feedback on the performance of the provider(s)
E	Local opinion leaders	Use of providers nominated by their colleagues as ‘educationally influential’. The investigators must have explicitly stated that their colleagues identified the opinion leaders
F	Patient-mediated interventions	New clinical information (not previously available) collected directly from patients and given to the provider, for example, depression scores from an instrument
G	Audit and feedback	Any summary of clinical performance of healthcare over a specified period of time. The summary may also have included recommendations for clinical action. The information may have been obtained from medical records, databases or patient observations
H	Reminders	The patient or provider encounters specific information designed or intended to prompt a health professional to recall information or perform or avoid some action to aid individual patient care. Computer-aided decision support is included
I	Marketing	Use of personal interviewing, group discussion (‘focus groups’) or a survey of targeted providers to identify barriers to change and subsequent design of an intervention that addresses identified barriers
J	Mass media	Either (1) varied use of communication that reached great numbers of people including television, radio, newspapers, posters, leaflets and booklets, alone or in conjunction with other interventions, or (2) targeted at the population level

EPOC, Effective Practice and Organisation of Care.

In this paper, we present an overview of systematic reviews of professional behaviour change interventions that addresses two key questions. First, we ask *what are the characteristics of relatively successful behaviour change interventions*? Second, we ask, *why are these characteristics important*? We examine the behaviour change literature through the lens of normalisation process theory (NPT).^10–12^ NPT focuses on action—the things that people do when they implement a new or modified way of conceptualising, enacting or organising practice, including the collective action that results from complex patterns of social relations and interactions^13^—rather than on their beliefs, attitudes and intentions. NPT characterises implementation processes as the product of four social mechanisms (see table 2): coherence (what users do to make sense of new practices); cognitive participation (what users do to engage with new practice); collective action (what users do to enact a new practice); and reflexive monitoring (what users do to appraise the effects of a new practice), and in doing so it facilitates an understanding of the contexts, social structure and processes through which behaviour change interventions are enacted.

**Table 2:** The constructs of NPT (adapted from^59^)

Group	Construct	Description	Code
Coherence	**Differentiation**	An important element of sense-making work is to understand how a set of practices and their objects are different from each other	CODI
	**Communal specification**	Sense-making relies on people working together to build a shared understanding of the aims, objectives and expected benefits of a set of practices	COCS
	**Individual specification**	Sense-making has an individual component too. Here participants in coherence work need to do things that will help them understand their specific tasks and responsibilities around a set of practices	COIS
	**Internalisation**	Finally, sense-making involves people in work that is about understanding the value, benefits and importance of a set of practices	COIN
Cognitive Participation	**Initiation**	When a set of practices is new or modified, a core problem is whether or not key participants are working to drive them forward	CPIN
	**Enrolment**	Participants may need to organise or reorganise themselves and others in order to collectively contribute to the work involved in new practices. This is complex work that may involve rethinking individual and group relationships between people and things	CPEN
	**Legitimation**	An important component of relational work around participation is the work of ensuring that other participants believe it is right for them to be involved, and that they can make a valid contribution to it	CPLE
	**Activation**	Once it is underway, participants need to collectively define the actions and procedures needed to sustain a practice and to stay involved	CPAC
Collective Action	**Interactional workability**	This refers to the interactional work that people do with each other, with artefacts, and with other elements of a set of practices, when they seek to operationalise them in everyday settings	CAIW
	**Relational integration**	This refers to the knowledge work that people do to build accountability and maintain confidence in a set of practices and in each other as they use them	CARI
	**Skill set workability**	This refers to the allocation work that underpins the division of labour that is built up around a set of practices as they are operationalised in the real world	CASW
	**Contextual integration**	This refers to the resource work—managing a set of practices through the allocation of different kinds of resources and the execution of protocols, policies and procedures	CACI
Reflexive Monitoring	**Systematisation**	Participants in any set of practices may seek to determine how effective and useful it is for them and for others, and this involves the work of collecting information in a variety of ways	RMSY
	**Communal appraisal**	Participants work together—sometimes in formal collaboratives, sometimes in informal groups to evaluate the worth of a set of practices. They may use many different means to do this drawing on a variety of experiential and systematised information	RMCA
	**Individual appraisal**	Participants in a new set of practices also work experientially as individuals to appraise its effects on them and the contexts in which they are set. From this work stem actions through which individuals express their personal relationships to new technologies or complex interventions	RMIA
	**Reconfiguration**	Appraisal work by individuals or groups may lead to attempts to redefine procedures or modify practices—and even to change the shape of a new technology itself	RMRE

EPOC, Effective Practice and Organisation of Care; NPT, normalisation process theory.

NPT has been previously been applied as a framework for theoretical analysis to qualitative systematic reviews of studies of the implementation of e-health systems;^14^ organisational change in healthcare provision for adolescents;^15^ professional behaviour around implementing guidelines^13^ and advance care plans;^16^ and patient help-seeking and self-care behaviours.^17^ Theory-led reviews using such frameworks offer opportunities to understand social mechanisms by which interventions work, rather than evaluating intervention effectiveness, which is our objective in this paper.

## Methods

### Inclusion and exclusion criteria

To be included, reports had to be peer-reviewed English language reports of systematic reviews, meta-analyses or syntheses of published qualitative or quantitative studies, that examined the effectiveness of interventions intended to lead to the implementation of evidence-based practice by healthcare professionals or providers, with the interventions evaluated being those defined as ‘Professional Interventions’ by the Cochrane Effective Practice and Organisation of Care review group.^2^ Comparisons of implementation intervention versus control (no intervention) or another intervention were acceptable. Included studies had to report any measures of clinical process change, compliance or patient outcomes. Reports were excluded if they focused on macrolevel organisational and policy changes in healthcare systems or evaluated public health or patient behaviour programmes (eg, smoking cessation and other lifestyle changes). Studies of the role of financial incentives in promoting behaviour change were excluded because these tend to be aimed at relatively autonomous professionals in fee for service environments, rather than complex workgroups in complex organisational settings. Studies which looked at the barriers or factors affecting implementation, rather than the effects of interventions themselves on outcomes, were also excluded. A copy of the protocol used for the review has been published online.^18^

### Searches and information sources

A literature search was carried out using the key words and search strategy detailed in box 1. Montori's^19^ optimal search strategy for maximum precision for retrieving systematic reviews from Medline was used. Also, given the close relationship between guideline implementation, practice patterns, evidence-based medicine and quality improvement, the search was broadened to include these Medical Subject Heading (MeSH) terms. The electronic databases MEDLINE (1947 to Present), CINAHL (1981 to Present), PsychINFO (1967 to present) were searched using EBSCO. In addition, the Cochrane library (1988 to present) was searched using the same search strategy outlined in box 1, adapted for use in the web interface. Citation and reference searching wasperformed on the articles selected for review. The last search was run in July 2015.

Box 1 **Search strategy used in overview of systematic reviews**

1. ‘clinicians’
2. (MH ‘Nurse Practitioners+)’ OR (MH ‘General Practitioners)’ OR ‘practitioner’
3. (MH ‘Nursing Staff+)’ OR (MH ‘Medical Staff+)’ OR (MH ‘Nursing Staff, Hospital)’ OR (MH ‘Medical Staff, Hospital+)’ OR ‘staff’
4. ‘health professional’ OR ‘health professionals’
5. ‘healthcare teams’ OR (MH ‘Patient Care Team+)’
6. (MH ‘Health Personnel)’ OR ‘health personnel’ OR (MH ‘Allied Health Personnel+)’
7. (MH ‘Allied Health Occupations+)’ OR (MH ‘Allied Health Personnel)’ OR ‘allied health professionals’
8. ‘occupational therapists’
9. (MH ‘Pharmacists)’ OR ‘pharmacist’
10. (MH ‘Nutritionists)’ OR ‘dietitians’
11. (MH ‘Physical Therapists)’ OR ‘physiotherapist’
12. (MH ‘Nurses+)’ OR ‘nurses’
13. (MH ‘Physicians)’ OR ‘physicians’
14. ‘doctors’
15. (MH ‘Algorithms+)’ OR ‘algorithm*’
16. (MH ‘Information Dissemination)’ OR ‘information dissemination’
17. (MH ‘Clinical Protocols+)’ OR ‘protocol’
18. (MH ‘Mass Media+)’ OR ‘mass media’
19. (MH ‘Medical Audit+)’ OR (MH ‘Nursing Audit)’ OR ‘audit’
20. (MH ‘Marketing+)’ OR ‘marketing’
21. ‘opinion leaders’
22. (MH ‘Reminder Systems)’ OR ‘reminder’
23. ‘academic detailing’
24. ‘educational outreach’
25. ‘educational materials’
26. (MH ‘Guideline+)’ OR ‘guideline’ OR (MH ‘Practice Guideline)’
27. (MH ‘Education+)’ OR ‘education’
28. ‘printed’
29. ‘identify barriers’
30. ‘reminders’
31. (MH ‘Process Assessment (Health Care))’ OR ‘process’
32. ‘outcomes’ OR (MH ‘Outcome Assessment (Health Care)+)’
33. (MH ‘Guideline Adherence)’
34. ‘behaviour’
35. (MH ‘Behavior+)’ OR ‘behavior’
36. (MH ‘Physician's Practice Patterns)’ OR (MH ‘Professional Practice+)’ OR (MH ‘Nursing, Practical)’ OR ‘practice’
37. ‘process of care’ OR ‘processes of care’ OR ‘health outcomes’ OR ‘patient outcomes’
38. AB MEDLINE OR TI MEDLINE OR AB systematic review OR TI systematic review OR PT meta-analysis
39. 1 OR 2 OR 3 OR 4 OR 5 OR 6 OR 7 OR 8 OR 9 OR 10 OR 11 OR 12 OR 13 OR 14
40. 15 OR 16 OR 17 OR 18 OR 19 OR 20 OR 21 OR 22OR 23 OR 24 OR 25 OR 26 OR 27 OR 28 OR 29 OR 30
41. 31 OR 32 OR 33 OR 34 OR 35 OR 36 OR 37
42. 38 AND 39 AND 40 AND 41

AB, abstract; MH, Medical Subject Heading; PT, publication type, ‘+’ indicates an exploded term; TI, title.

### Study selection

Studies were assessed for eligibility by both reviewers, who were not blinded to the identities of the study authors or institutions.

### Data collection process

Data extraction was carried out by a single author (MJJ) working alone and using a data extraction instrument that encompassed the subject of the review, the setting, the participants, the intervention assessed, the outcome measures, the years of literature searched, the main findings and authors’ conclusions. Reviews were then coded to which interventions they included by two reviewers working together, using the full manuscript of each review.

### Quality assessment of included systematic reviews

The quality of included reviews was assessed using the AMSTAR criteria.^20^ Studies scored one point for each of the 11 criteria they met, and scored 0 if they did not meet the criteria or it could not be assessed due to a lack of reported information (see online supplementary file A for more details).

### Synthesis of results

This is an overview of systematic reviews, so vote counting together with a narrative synthesis of included studies was planned to summarise findings. This was because some meta-analysis may have already taken place in the included studies; the likelihood of varying areas of focus between reviews; and anticipated heterogeneity in the reporting of results. Systematic reviews which focused specifically on guideline implementation as an activity were analysed separately. Where a systematic review had included studies that used more than one kind of intervention, it was considered to be assessing multiple strategies. For the purpose of synthesis, systematic reviews considering multiple intervention types were coded to each of the intervention types they assessed, with effectiveness of their component interventions being assessed individually. This strategy meant that the studies included in several reviews would be counted more than once, but helped gauge the effectiveness of each intervention type when used as part of a multifaceted strategy.

### Mapping of EPOC professional interventions to NPT

Both authors mapped each of the 10 intervention types (excluding the ‘Other’ category), defined by EPOC (see table 1) to 14 of the 16 subconstructs of NPT (see table 2), and developed a coding matrix incorporating both NPT constructs and EPOC intervention types. We excluded two NPT subconstructs from coding: differentiation and reconfiguration, because the first is a precondition for an experimental intervention and the second is a normal requirement of an intervention study.

### Coding of systematic reviews to NPT framework

Once included, systematic reviews were assigned to one of three groups: those considering guideline implementation, those considering single interventions, and those which considered studies using multiple interventions. Reviews were coded as using single interventions if they considered only one type of professional intervention exclusively, while those that included studies using a variety of interventions or combinations of interventions were coded as using multiple interventions. Each systematic review was then coded using framework analysis, as to which interventions it used (based on the studies it had included), and the NPT-EPOC professional intervention coding framework then used to determine which NPT constructs it had covered in its component interventions. This then allowed each review to be given a score for each construct of NPT depending on which EPOC intervention type had been used in the included studies when drawing conclusions about effectiveness. Each systematic review was then also coded as to whether it had concluded that the intervention/interventions it had reviewed had been successful in improving the process of care and/or patient outcomes. For each of these two outcomes, systematic reviews could be coded as ‘successful’, ‘unsuccessful’ or ‘not assessed’. Reviews where authors concluded that effectiveness could not be determined, or where results presented were mixed, were coded as ‘unclear’. This was in essence a qualitative framework analysis presented using simple counts.^21^ ^22^

## Results

### Results of searches

**Figure 1:**
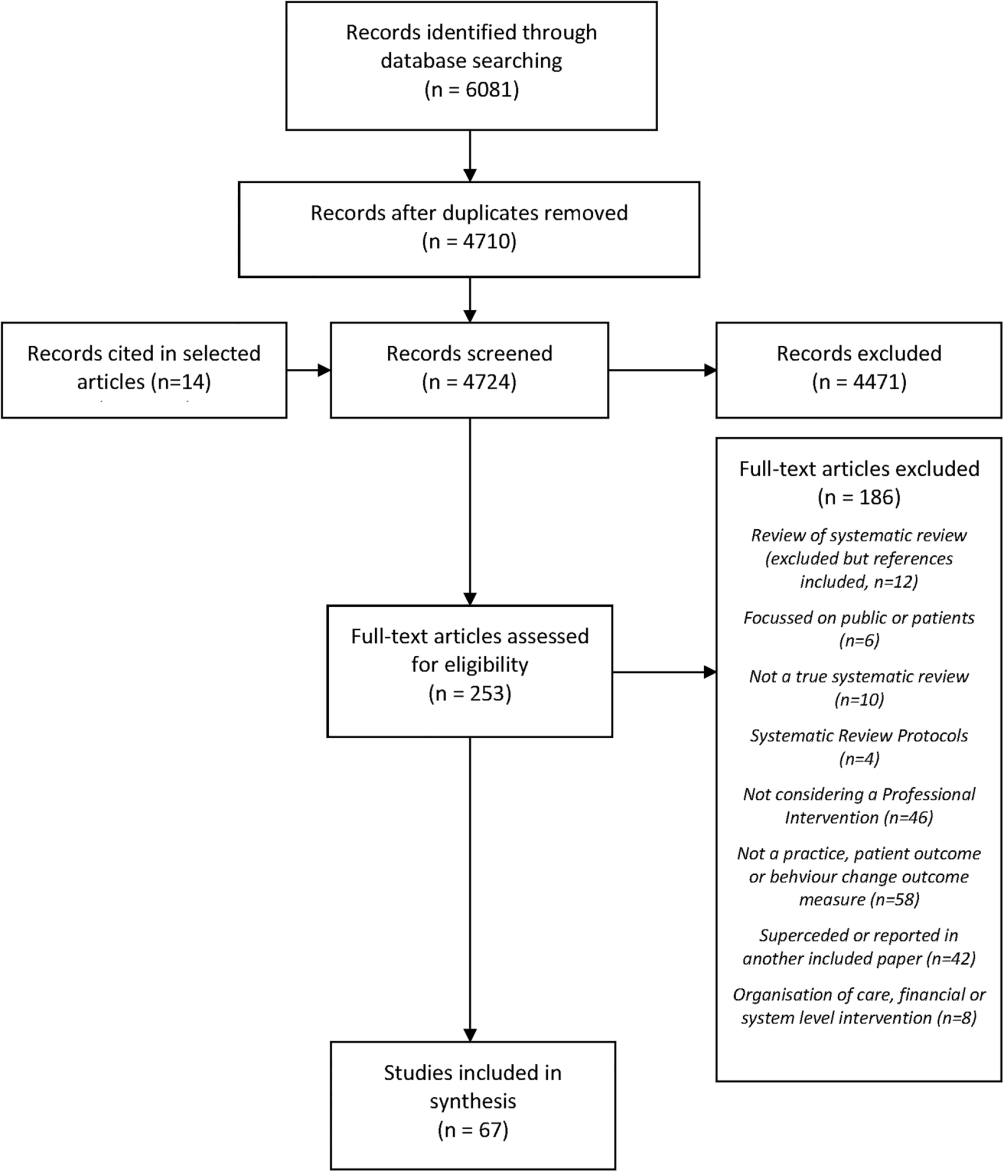
Flow chart of systematic review process.

We describe the review process in figure 1. We identified 6081 possible articles, with 4710 left after removal of duplicates. A further 14 were cited by selected articles, meaning that 4724 entered the first stage of the review process; 253/4724 were selected for review of the full text; and 67/253 fully met the criteria for inclusion. Of these, 20/67 focused on primary, ambulatory or community care; 11/67 focused on secondary or specialist care, and 36/67 focused on both primary and secondary care settings. Included reviews fell into three groups: 34/67 reviewed studies of a single type of intervention (see table 3); 33/67 reviewed studies of multiple types of intervention. Of the latter, 21/33 considered multifaceted interventions aimed at improving practice or patient outcomes (see table 4), while 12/33 specifically examined guideline intervention strategies. These were considered separately (see below and table 5). The findings are considered in more detail below using the EPOC PI classification. Details of all included studies can be found in attached online supplementary file B. The strategies used in included studies fell into three main categories: persuasive interventions; educational and informational interventions; and action and monitoring.

**Table 3:** Summary: effectiveness of single interventions

Intervention focus	Intervention type	Total number of reviews (Mean quality score)	Professional practice				Patient outcome			
			n	Effective (%)	Ineffective (%)	Unclear (%)	n	Effective (%)	Ineffective (%)	Unclear (%)
Persuasion	Marketing	1 (11)	1	1 (100)	0 (0)	0 (0)	0	–	–	–
	Mass media	0 (NA)					0	–	–	–
	Local consensus processes	0 (NA)	0	–	–	–	0	–	–	–
	Local opinion leaders	1 (10)	1	1 (100)	0 (0)	0 (0)	0	–	–	–
Education	Patient-mediated interventions	0 (NA)	0	–	–	–	0			
	Distribution of educational materials	6 (8.3)	5	3 (60)	1 (20)	1 (20)	5	2 (40)	1 (20)	2 (40)
	Educational meetings	5 (8)	4	3 (60)	1 (20)	1 (20)	2	1 (50)	0 (0)	1 (50)
	Educational outreach	2 (8.5)	2	2 (100)	0 (0)	0 (0)	1	0 (0)	0 (0)	1 (100)
Action	Audit and feedback	1 (10)	2	1 (100)	0 (0)	0 (0)	1	1 (100)	0 (0)	0 (0)
	Reminders	18 (7.6)	18	14 (78)	2 (11)	2 (11)	11	4 (36)	2 (18)	5 (45)

NA, not applicable.

**Table 4:** Summary: effectiveness of multifaceted interventions

Intervention focus	Intervention type	Total number of reviews (Mean quality score)	Professional practice				Patient outcome			
			n	Effective (%)	Ineffective (%)	Unclear (%)	n	Effective (%)	Ineffective (%)	Unclear (%)
Persuasion	Marketing	4 (8)	4	2 (50)	0 (0)	2 (50)	2	0 (0)	0 (0)	2 (100)
	Mass media	2 (9)	2	0 (0)	0 (0)	2 (100)	2	0 (0)	0 (0)	2 (100)
	Local consensus processes	2 (7.5)	2	0 (0)	0 (0)	2 (100)	1	0 (0)	0 (0)	1 (100)
	Local opinion leaders	4 (7)	4	2 (50)	1 (25)	1 (25)	2	0 (0)	1 (50)	1 (50)
Education	Patient-mediated interventions	4 (8.3)	4	3 (75)	0 (0)	1 (33)	2	1 (50)	0 (0)	1 (50)
	Distribution of educational materials	15 (8.3)	15	11 (73)	1 (7)	3 (20)	11	5 (45)	2 (18)	4 (36)
	Educational meetings	16 (7.8)	16	11 (69)	0 (0)	5 (31)	8	2 (25)	1 (13)	5 (63)
	Educational outreach	12 (7.6)	12	8 (67)	1 (8)	3 (25)	7	1 (14)	2 (29)	4 (57)
Action	Audit and feedback	15 (8)	15	12 (80)	0 (0)	3 (20)	6	2 (33)	1 (17)	3 (50)
	Reminders	15 (7.1)	15	11 (73))	1 (7)	3 (20)	7	1 (14)	2 (29)	4 (57)

**Table 5:** Summary: guideline implementation strategies

Intervention focus	Intervention type	Total number of reviews (Mean quality score)	Professional practice				Patient outcome			
			n	Effective (%)	Ineffective (%)	Unclear (%)	n	Effective (%)	Ineffective (%)	Unclear (%)
Persuasion	Marketing	4 (6.8)	4	3 (75)	0 (0))	1 (25)	2	2 (100)	0 (0)	0 (0)
	Mass media	2 (7.5)	2	2 (100)	0 (0)	0 (0)	1	1 (100)	0 (0)	0 (0)
	Local consensus processes	2 (7.5)	2	2 (100)	0 (0)	0 (0)	1	1 (100)	0 (0)	0 (0)
	Local opinion leaders	5 (6.2)	5	5 (100)	0 (0)	0 (0)	2	2 (100)	0 (0)	0 (0)
Education and Information	Patient-mediated interventions	3 (7.3)	3	3 (100)	0 (0)	0 (0)	1	1 (100)	0 (0)	0 (0)
	Distribution of educational materials	NA	NA	NA	NA	NA	NA	NA	NA	NA
	Educational meetings	8 (6.3)	8	6 (75)	0 (10)	2 (25)	5	4 (80)	0 (0)	1 (20)
	Educational outreach	7 (6.7)	7	6 (86)	0 (0)	1 (14)	4	4 (100)	0 (0)	0 (0)
Action	Audit and feedback	9 (6.3)	9	7 (78)	0 (0)	2 (12)	5	4 (80)	0 (0)	1 (20)
	Reminders	12 (6.7)	12	9 (75)	1 (8)	2 (17)	7	5 (71)	1 (14)	1 (14)

NA, not applicable.

### Quality assessment

The quality score was generally lower for studies looking at different guideline implementation strategies (mean score 6.7) than those considering single interventions (see tables 3 and 4), overall mean scores of 8 and 7.5 for multiple intervention reviews and single professional intervention reviews, respectively, see online supplementary file A). Low scores appear to be mainly due to inadequate reporting. Many studies failed to assess publication bias (82%) or include a list of included and excluded publications (69%).

### Persuasive interventions

Some behaviour change strategies rely on persuasion and offer participants high levels of discretion over the means by which behavioural change is enacted. Diffuse persuasive strategies include *Marketing* and *Mass Media* approaches. Oxman *et al*^23^ suggested that while marketing was important in targeting interventions, it was not possible to separate its effect from other interventions. Baker *et al*^24^ concurred, though he noted that tailoring interventions to prospectively identified barriers was more likely to improve practice than not. Four reviews looking at multifaceted interventions considered marketing, with two finding benefits to professional practice, though the effect on patient outcomes was mixed.^23^ ^25–27^ Direct persuasion includes approaches that build on and exploit *Local Consensus Processes* and *Local Opinion Leaders.* Only two reviews of multifaceted interventions considered local consensus processes, but neither showed clear improvements in practice or patient outcomes.^23^ ^28^ Flodgren *et al*^29^ found that local opinion leaders had a positive effect on professional behaviour change. However, they noted that the role of opinion leaders is poorly defined, making it difficult to ascertain the optimal approach to this particular intervention. Four systematic reviews included studies using local opinion leaders as part of multifaceted interventions, and had inconsistent and ambiguous findings.^23^ ^27^ ^30^ ^31^

### Educational and informational interventions

These focus on the availability of educational materials and other types of clinical information. *Patient-Mediated Interventions* offer health professionals new clinical information collected directly from the patient. No reviews considered patient-mediated interventions in isolation from other strategies, although four considered multifaceted interventions that included them. Oxman *et al*'s^23^ early review emphasised uncertainty about their effectiveness. More recently, French *et al*^32^ have found that such interventions had potential for benefit in imaging for musculoskeletal conditions. Davis *et al*^30^ and Brennan *et al*^27^ also found benefits to practice in their reviews.

Six reviews focused solely on the *Dissemination of Educational Materials*; Thomas *et al*^33^ and Giguère *et al*^34^ concluded that printed materials had a positive effect on professional practice, but an unclear effect on patient outcomes. Blackwood *et al*^35^ found positive effects on weaning in ventilated patients in intensive care; and Clarke *et al*^36^ found benefits to practice in surgical referral using guidelines. Worrall *et al*'s^37^ earlier review and Wutoh *et al*'s^38^ more recent one found no clear benefit to practice in primary care. Where educational materials were part of multifaceted interventions, 11/15 studies showed a benefit to the process of care or practice, and 5/11 found a benefit to patient outcomes. Goodwin *et al*^39^ and Forsetlund *et al*^40^ found evidence of positive effects of *Educational Meetings* on professional behaviour, and Forsetlund *et al* also found some benefit to patient outcomes. Brody *et al*^41^ also found that participation in education meetings improved management of dementia. While there were benefits to practice from educational meetings, the effects on patient outcomes were less clear, with just two studies^40^ ^41^ focusing on them in isolation. Educational meetings were considered by 16 reviews looking at multifaceted interventions in improving professional practice, and were found to be effective in 11/16 reviews, with just two finding a benefit for patients.^32^ ^42^

O'Brien *et al*^43^ showed that *Educational Outreach* (also known as academic detailing) is effective in changing practice, though the effect size varied depending on the clinical domain, as did Chhina *et al*'s^44^ more recent review. Twelve reviews considering multiple intervention types looked at educational outreach, with 8/12 finding them effective in changing practice. Two reviews asserted that educational outreach interventions using academic detailing are superior to other intervention types.^30^ ^45^

### Action and monitoring

Other behaviour change interventions seek to shape clinical practice by continuously monitoring and reinforcing desired behaviours. In their important review, Ivers *et al*^46^ found that *Audit and Feedback* lead to improvements in professional practice and patient outcomes, though the effect sizes were often small but potentially important. Effectiveness depended on baseline measures and the method for delivering feedback. Eleven reviews of multifaceted interventions found benefits to professional practice from audit and feedback. Eighteen reviews looked at *Reminders* alone, including the eight that focused on the use of computer-based clinical decision support systems (CDSS), two that focused on computerised information systems and eight that investigated computerised or paper-based reminders. Fourteen of the eighteen reviews provided evidence suggesting that reminder based systems are beneficial in improving the process of care. Of the four that did not show clear benefit, three focused on general CDSS rather than specific reminders or prompts.^47–49^ Only 4 of the 11 which reported an effect on patient outcomes found a positive effect.^50–53^ Fifteen of the studies that reviewed multifaceted professional interventions considered reminders, with 11/15 finding them to be effective in improving professional practice. Six of the seven reviews which considered patient outcomes were unclear about their effectiveness, with a benefit seen in just one review.

### Guideline implementation strategies

Twelve systematic reviews specifically considered optimal strategies for guideline implementation, and we evaluate those separately in this section (they have not been considered elsewhere in this review). Seven of the reviews that addressed guideline implementation strategies compared in some way various single implementation strategies with multifaceted approaches which used a combination of interventions. Grimshaw *et al* in 2004^54^ showed no difference between single and multifaceted strategies, a finding also confirmed by Hakkennes and Dodd in 2008.^55^ However, a more recent systematic review by Medves *et al*^56^ found a benefit of multifaceted strategies, particularly for more complex healthcare areas. They suggest that interventions that link local opinion leaders, audit and feedback and reminders were the most effective strategies. Chaillet *et al*^57^ also concluded that multifaceted strategies based on audit and feedback, perhaps facilitated by local opinion leaders, appeared most effective in an obstetric setting. Table 5 shows that, when used as part of guideline implementation strategies, most professional interventions were effective at improving practice and patient outcomes. The most frequently studied interventions were educational meetings, audit and feedback, reminders, educational outreach visits and local opinion leaders. Three reviews examining implementation strategies drew attention to the need to identify barriers to implementation, and to tailor implementation strategies to their settings.^55^ ^57^ ^58^ In particular, Chaillet *et al*^57^ noted that interventions where barriers to change were prospectively identified were more likely to be successful (93.8% vs 47.1%, p=0.04).

### Mapping EPOC to NPT

The NPT-EPOC framework that was developed is shown in table 6. This shows that the EPOC intervention types which act across the greatest number of NPT constructs are *Audit and Feedback*, *Reminders* and *Educational Outreach*. The order of the professional interventions in table 6 is based on how effective they are at changing professional practice according to the overall findings presented above, taking tables 3, 4 and 5 together, with each of the 10 professional intervention types ranked in order from 1 to 10, with the most effective at the top of the table and least effective at the bottom. It can be seen that more effective interventions tend to act across more NPT constructs, but in particular are those that act in the areas of *Collective Action* and *Reflexive Monitoring*. Less effective interventions tend to focus on *Coherence* or the early stages of *Cognitive Participation* alone.

**Table 6:** NPT-EPOC professional Intervention coding framework

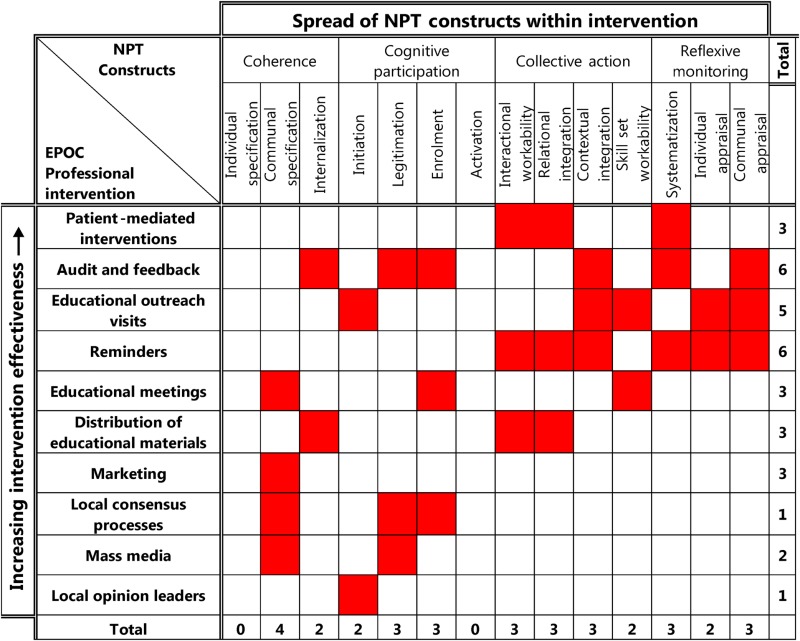

Interventions have been ranked in order of effectiveness in changing professional practice according to the findings of this overview. The NPT constructs acted on by each intervention are highlighted in red.

EPOC, Effective Practice and Organisation of Care; NPT, normalization process theory.

## Discussion

This theory-led overview of systematic reviews has demonstrated that interventions based on action (such as audit and feedback, and reminders) and various types of education tend to be more likely to successfully change professional behaviour than those based on persuasion, such as local consensus processes and opinion leaders. Interventions more likely to be successful seem to act through the NPT constructs of *Collective Action* and *Reflexive Monitoring*.

### Limitations of the overview

Overviews of systematic reviews are subject to important limitations, especially when they deal with interventions that are heterogeneous, complex and non-standardised. In this overview, we found great variability in the effect size seen within each intervention considered. This was almost certainly further complicated by the effects of methodological advances over the past 30 years. This means that while we can describe findings in general indicative terms, we cannot draw definitive conclusions about effectiveness. This was exacerbated by problems of reporting. Some studies claimed to review single intervention types but actually included studies containing bundles of interventions. This is unsurprising because most attempts to change behaviour involve bundles of interventions. However, it means that the results of these reviews may have been clouded by unconsidered components in the studies included. The complex nature of professional interventions is similarly a problem when assessing effectiveness. Several reviewers pointed out the difficulties and frustrations associated with trying to ‘pick apart’ which components of complex interventions were their ‘active ingredients’, and were forced to conclude that it was not possible to clearly assess the effectiveness of particular components. One of the reasons for choosing to perform an overview of systematic reviews rather than a standard systematic review was to try to capture an overarching sense of which interventions and combination of interventions seemed to be successful in the context of this complexity. The reviews in this overview were spread across a wide range of settings, so again general conclusions should be drawn with caution. Publication bias may be an important problem in this body of literature since it suggests that most intervention types have a positive effect on measures of process or professional behaviour (such as compliance with a guideline or use of a particular resource), but is less certain about effects on patient outcomes.

This overview has used the Cochrane EPOC taxonomy of behaviour change interventions as a framework to consider the different interventions and strategies. However, while it is convenient to classify interventions in this way, particularly when reviewing groups of interventions, in reality most interventions aimed at individuals or social groups are much more complex, with a single intervention often sharing elements with others in a separate classification. The EPOC taxonomy can therefore be quite a blunt instrument when trying to understand interventions in complex healthcare settings.

### What are the characteristics of relatively successful professional behaviour change interventions?

The limitations of a review like this act as important deterrents against definitive conclusions about what kinds of interventions are most effective. Our approach is somewhat different. By using a theory of practice as the lens through which data are interpreted, we seek to suggest explanations for the underlying processes by which interventions have their effects, highlighting key elements which seem to be important in successful professional practice change. Our approach also suggests why bundles of interventions packaged together seem more effective than single interventions. This is not because they have an aggregate or cumulative effect, but because they link together to form social systems that promote changes in behaviour norms. This means that the collective rather than individual action constructs of NPT explain key components of effective behaviour change interventions. If this is true, it may explain the preponderance of negative trials of behaviour change interventions founded on models of individual intentions and behaviours.

NPT helps us to gain some insight into why some interventions appear more effective than others. Table 6 shows that the least effective interventions focus on work that invests in clinicians’ coherence (how they make sense of what the intervention asks of them) and cognitive participation at the expense of collective action (what they actually do) and reflexive monitoring (how they appraise the effects of their actions). In contrast, the most effective interventions (Educational Outreach using Academic Detailing, Audit and Feedback, and Reminders) call for coherence but also emphasise collective action and reflexive monitoring. These interventions provide mechanisms for participants to relate their *performance* to external reference group expectations, opportunities for revealing and reinforcing internal peer group norms, and for these mechanisms to operate continuously over time. In other words, participants in successful behaviour change interventions may have responded positively to a clear sense of how what they were asked to do made sense (its coherence), and how their actual responses to this (their collective action) measured up to the expectations of external observers (reflexive monitoring). In the case of guideline implementation studies, this process also seems to include a need for additional investment in cognitive participation: in particular, investment devoted to overcoming questions about the legitimacy of new guidelines and the need to enrol clinicians into their use. This suggests that behaviour change follows changes in structure and action rather than it being the product of changes in beliefs and intentions.

## Conclusion

This is the first overview of systematic reviews to use NPT to guide analysis. The limitations that we have described above mean that we must be cautious in the empirical claims that we make about the degree of effectiveness that is attached to particular intervention types. However, in general terms, we are able to sketch a conceptual model of their actions, and represent these as hypotheses. Our first hypothesis is that:

- *Hypothesis 1*. Interventions that seek to restructure and reinforce new practice norms and associate them with peer and reference group behaviours are more likely to lead to behaviour change.

Two kinds of interventions contribute to the processes proposed in Hypothesis 1: (1) normative restructuring of practice modifies peer group expectations of practice (eg, opinion leaders, educational outreach, educational meeting and materials/guidelines); and (2) relational restructuring reinforces modified peer group norms by emphasising the expectations of an external reference group (eg, Educational Outreach using Academic detailing, Reminders, Audit and Feedback). Bundled together, such interventions create a coherent and legitimised set of rules about the conduct of practice; where enacting those rules is made to become a normal component of everyday work; and where individual participants are encouraged to replicate activities common to their peers. Importantly, such interventions tend to use action or education, and focus on *Collective Action* and *Reflexive Monitoring.* Our second hypothesis supports this by highlighting outcomes of interventions that have ‘soft’ attitudinal components:

- *Hypothesis 2*. Interventions that seek to reshape the attitudinal landscape in which professional behaviours are enacted are less likely to lead to behaviour change.

Importantly, the kinds of interventions specified by Hypothesis 1 are ones that operationalise clear mechanisms that shape behaviour norms—rules that give structure to everyday actions. However, the interventions that contribute to the process defined in Hypothesis 2 are characterised by more diffuse mechanisms: (1) indirect attempts to redefine behaviours and the scope of practice (eg, marketing and mass media campaigns); and (2) local attempts to reformulate ideas about practice (eg, consensus building exercises). Such interventions tend to use persuasion rather than action, and are likely to focus more on understanding (*Coherence*) and the early stages of *Cognitive Participation*.

Our overview of systematic reviews suggests that successful behaviour change interventions operationalised in complex organisational environments are likely to require intervention types that lead to normative and relational restructuring (and hence a focus on collective rather than individual action), and the legitimation of new practice norms through experience. Further research is required to develop and test these hypotheses and to assess the utility of the theoretical model that we propose here.
